# Assessment of the Nurse Medication Administration Workflow Process

**DOI:** 10.1155/2016/6823185

**Published:** 2016-07-17

**Authors:** Nathan Huynh, Rita Snyder, José M. Vidal, Omor Sharif, Bo Cai, Bridgette Parsons, Kevin Bennett

**Affiliations:** ^1^Civil & Environmental Engineering, University of South Carolina, Columbia, SC 29208, USA; ^2^College of Nursing, University of Arizona, Tucson, AZ 85721, USA; ^3^Computer Science and Engineering, University of South Carolina, Columbia, SC 29208, USA; ^4^Epidemiology and Biostatistics, University of South Carolina, Columbia, SC 29208, USA; ^5^School of Medicine, University of South Carolina, Columbia, SC 29208, USA

## Abstract

This paper presents findings of an observational study of the Registered Nurse (RN) Medication Administration Process (MAP) conducted on two comparable medical units in a large urban tertiary care medical center in Columbia, South Carolina. A total of 305 individual MAP observations were recorded over a 6-week period with an average of 5 MAP observations per RN participant for both clinical units. A key MAP variation was identified in terms of unbundled versus bundled MAP performance. In the unbundled workflow, an RN engages in the MAP by performing only MAP tasks during a care episode. In the bundled workflow, an RN completes medication administration along with other patient care responsibilities during the care episode. Using a discrete-event simulation model, this paper addresses the difference between unbundled and bundled workflow and their effects on simulated redesign interventions.

## 1. Introduction

In recent years, concern about the impact of health care system interventions on clinical workflow processes has escalated primarily due to the implementation of electronic health records and computerized provider order entry systems [1–4]. The impact and unintended consequences of these system redesign interventions have underscored the lack of knowledge about high-risk clinical processes and the concomitant patient safety risks associated with system redesign that may destabilize these processes in dynamic care delivery environments [1]. Evidence about clinical workflow processes is quite limited. Their dynamic nature and the complexity of healthcare environments within which they occur make them difficult to assess with current observation methods and tools. There is a critical need for innovative methods and technologies that support a low-risk environment in which to visualize, examine, and manipulate high-risk clinical processes to assess the potential impact of redesign interventions on multilevel systems, clinician, and patient outcomes [5, 6].

Medication errors remain a serious health care problem in the United States which result in approximately 7,000 deaths, cause harm to approximately 1.5 million people, and cost billions of dollars in hospital treatment annually [7–11]. Leape et al.'s [12] early research into medication errors highlighted the need to examine system factors associated with medication errors. This research used a systems analysis approach to examine all phases of the medication process, that is, physician ordering, transcription/verification, pharmacy dispensing, and nurse administration, to determine types of medication errors by stage of drug ordering and delivery. Findings indicated that, of the identified medication errors (*n* = 334), nurse medication administration errors (38%) were the second largest category of medication errors following physician medication order errors (39%) [12]. The MAP is, predominantly, a nursing responsibility that has been estimated to consume approximately 40% of nursing practice time [13]. Since Leape et al.'s [12] early research on medication errors, the MAP has become increasingly complex due to escalating patient acuity, numerous generic and trade medication names, expanded medication delivery routes, increased use of new and diverse medication safety technologies, and an increased number of medication orders [13, 14]. The lack of standardization of the MAP is also a key contributing factor in medication administration complexity [13].

Typically, the MAP involves a Registered Nurse (RN) performing many tasks, including, but not limited to, (1) assessing the patient to obtain pertinent data, (2) gathering medications, (3) confirming the six rights (i.e., right dose, patient, route, medication, time, and documentation), (4) administering the medications, (5) documenting administration, and (6) observing for therapeutic and untoward effects [15]. Clinical observation has confirmed a significant degree of nurse-to-nurse variability in MAP tasks and task sequencing that is subject to environmental interruptions that result in medication administration practices that deviate from standard practice protocols. Empirical evidence, however, is lacking about MAP characteristics and the nature of RN and environmental characteristics and interruptions that influence it. This makes it very difficult to anticipate the impact of system redesign interventions undertaken to enhance medication safety, for example, introduction of medication safety technology and the potential consequences of redesign interventions. To this end, this study identified MAP workflow characteristics and developed a computer simulation model based on these characteristics to assess the impact of simulated MAP redesign interventions on selected MAP redesign outcomes. It builds on our previous work where we developed a mobile application for recording live MAP observations [16] and a discrete-event simulation model of the MAP [17].

## 2. Methods

### 2.1. Setting

The study was undertaken in a tertiary medical center in Columbia, South Carolina, United States. The medical center is a 414-bed modern complex that anchors a comprehensive network of 600-plus affiliated physicians, including six strategically located community medical and urgent care centers, an occupational health center, the largest extended care facility in the Carolinas, and an Alzheimer's Care Center. The medical center also supports an array of health and wellness classes. The hospital employs more than 5,200 people and offers a variety of community outreach programs and education and health screenings. A high surgical volume is supported with 29 state-of-the-art operating rooms that have cutting edge, state-of-the-art medical technology and procedures. Patient safety and security are top concerns and health care team work is stressed. Identification bands, proper cough etiquette, frequent hand-washing, protective wear, and public safety officers are integral parts of the medical center's commitment to patient safety and security.

The study was conducted on two comparable medical units that served adult patients with medical, surgical, neurological, oncology, orthopedic, and renal conditions. Patients were typically admitted for chronic care management, diagnostic studies, and medical interventions. The units were comparable in terms of size (range = 30–36 beds), average daily census (range = 26–31 patients), patient length of stay (range = 6.5–7 days), and number of RNs (range = 25–29 full-time equivalent), as well as numbers and types of technical and secretarial support staff. The term “average” refers to arithmetic mean hereafter. Both clinical units comprised single bed rooms along 4 hallways. Each patient room had an individual bathroom, an inside supply cabinet, and a locked medication cabinet. A wall-mounted locked documentation station with a drop-down writing platform was located outside each patient room. A nurses' station was centrally located with an adjacent dumbwaiter and various separate rooms, for example, medications, clean/soiled equipment, supplies, breaks, family consultation, and a manager office. An integrated electronic health record (EHR) was used on each unit for all documentation, including medication administration and management. Two rolling computer carts were available on each hallway and RNs used them during walking rounds to receive change-of-shift report, and to document all care, including medication administration. More acutely ill patients were routinely assigned to rooms closer to the nurses' station.

### 2.2. Sample

Since medication administration is predominantly a responsibility of RNs, the study sample comprised RNs. The study did not involve patients and no patient information was collected during the course of the study. Following protocol approvals from university and medical center Institutional Review Boards, RN volunteers were recruited from full-time RN populations for each study unit. All full-time RNs from Unit 1 (*N* = 16) and Unit 2 (*N* = 20) were invited to participate. Study recruitment information was distributed at unit staff meetings by unit managers and during unit recruitment visits by the study Principal Investigator (PI). Registered Nurses interested in volunteering for the study met with the PI in a unit conference room where the PI explained the study prior to them signing a consent form and completing a demographic information form. A total of 17 RNs participated in the study with 7 from Unit 1 (44%) and 10 from Unit 2 (50%). Demographic findings indicated that RN characteristics were comparable across the two units. Combined findings for all 17 RN volunteers indicated that the majority were white [*n* = 15 (88%)] women [*n* = 16 (94%)] who had been licensed as an RN an average of 11 years, had practiced as an RN an average of 10 years, and had worked at the medical center an average of 6 years with 60% or more of that time spent on their study unit. The majority of RNs reported that they had a totally supportive medication safety culture [*n* = 11 (65%)] on their unit and that they felt totally comfortable [*n* = 10 (59%)] with reporting medication safety practice variations. Most RNs also indicated that they thought about medication safety frequently [*n* = 15 (88%)] and that the quality of the medication safety process on their unit was extremely high [*n* = 9 (53%)]. Medication safety technology, such as smart intravenous infusion pumps, was readily available [*n* = 13 (76%)] on the units, and the majority of the RNs used it frequently [*n* = 16 (94%)]. The majority of RNs had also completed 1 self-paced computer instructional program [*n* = 15 (88%)] on medication safety, with 7 (41%) completing this within one month prior to the study. The majority of RNs reported that they were very likely [*n* = 11 (65%)] to be interrupted while engaged in the medication administration process. Their self-reports, however, did not provide any specific interruption types or patterns.

### 2.3. MAP Observation Data Collection

#### 2.3.1. MAP Observation Recording Tool

**Figure 1:**
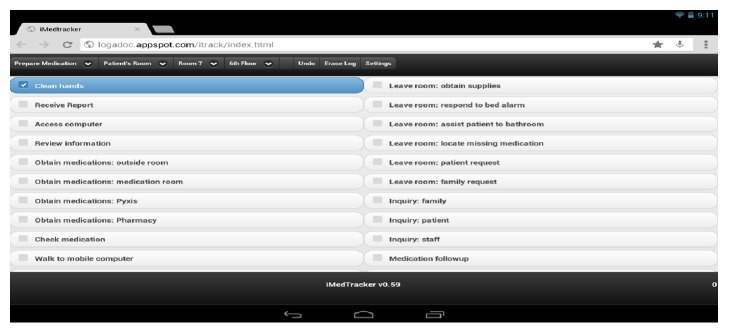
Screenshot of iMedTracker.

To record RN medication administration functions, tasks, and interruptions in a live clinical environment and to generate baseline data for the development of our computer simulation model, the research team developed the “iMedTracker” mobile application; it is an extension of the application we developed in our previous study [16]. The iMedTracker is a web-based application developed using the jQuery Mobile framework (http://jquerymobile.com/). The team chose to develop a web-based application instead of a native application because it would allow the application to run on any smartphone or tablet. The iMedTracker represents one of the first mobile applications designed specifically to record MAP workflows and one that can run on multiple mobile devices.  Figure 1 provides a screenshot of the iMedTracker application running on a Nexus 10.

The first menu item of iMedTracker is the MAP function. Observers can change the function by tapping on the item. When the item is pressed, a drop-down list is displayed for users to choose from. The list of choices include “prepare medication” and “administer medication.” There is a separate list of tasks associated with each function, and they change according to the function selected. To facilitate the recording process, the medication documentation tasks are listed under the administer medication function.

The three menu items to the right of “prepare medication” allow observers to record the location of the activity (e.g., patient's room, medication room, and kitchen), patient's room number, if applicable, and the clinical unit number (e.g., 6th and 7th). As their names imply, the “Undo” menu item undoes the last recorded activity, and the “Erase Log” menu item erases the stored data. The last menu item “Settings” takes observers to another screen where they can input their name, input the code (instead of name) of the RN being shadowed, and e-mail the data to project investigators. No patient data are recorded.

On the iMedTracker, the left column lists the tasks, and the right column lists the interruptions. When a task or interruption is selected, the application automatically captures its start time and writes the record to the database. The application indicates that the activity is in operation by highlighting that item, as illustrated by the “clean hands” task in Figure 1. To record the end of an activity, observers would press on that item and the application would automatically capture the activity's end time. If a mistake is made, observers can tap on the Undo button to cancel the last action.

#### 2.3.2. MAP Observation Data Collection Protocol

The MAP observational data collection protocol was field tested in this study using expanded methods and developed in our previous studies [16, 17]. Several steps were taken to ensure data recording consistency including (1) observer training in the use of the mobile device and iMedTracker application; (2) creation of common observation rules and data collection procedures; and (3) assessment of observer interrater reliability (IRR).

Three student observers participated in two 4-hour classroom training sessions that included (1) an introduction to the iMedTracker application, (2) group recording practice using iMedTracker and sample MAP videotapes from a previous study, and (3) four independent recording sessions using iMedTracker and sample MAP videotapes. Independent observer recordings were compared and discussed by the group to assess recording variations. Following classroom training, observers participated in a one-week orientation to the two study units. During this week, observers used iMedTracker to record live MAP observations for three data collection sessions and participate in debriefing conferences with study investigators at the conclusion of each session. Debriefing feedback was used to establish observation rules to enhance observer recording consistency and clarify data collection procedures (Table 1).

Interrater reliability was assessed 3 times during the 6-week data collection period. For each IRR assessment, a total of 4 separate MAP observations were recorded. Each assessment involved all three observers simultaneously recording a study RN's MAP task sequence for each of her/his assigned patients. Edit distance ratios (EDRs), using the Demareau-Levenshtein approach, were calculated to assess IRR [19]. Edit distance is derived from information theory and is specifically used to assess IRR with uneven task sequences. It represents the difference or distance between two strings of characters in terms of the number of edit operations, that is, insertion, deletion, and/or substitution, needed to transform the first string into the second one [20, 21]. All EDRs met the standard minimum 0.70 agreement criterion for multiobserver pairwise comparisons with Time 1 EDR means of 0.78, 0.69, and 0.69; Time 2 means of 0.80, 0.71, and 0.73; and Time 3 means of 0.86, 0.72, and 0.73 [22].

#### 2.3.3. MAP Observations

An observation was defined as an individual patient care episode that began with the first medication administration task performed and ended when the RN moved to the next patient. Trained student observers conducted a total of 54 MAP sessions over six weeks, during the hours of 7:00 to 11:00 AM on Mondays, Wednesdays, and Fridays in the months of June and July. A total of 305 individual MAP observations were recorded during the 54 MAP sessions with an average of 5 MAP observations per RN study participant for both clinical units.

### 2.4. Data Analysis

#### 2.4.1. Process Maps

**Figure 2:**
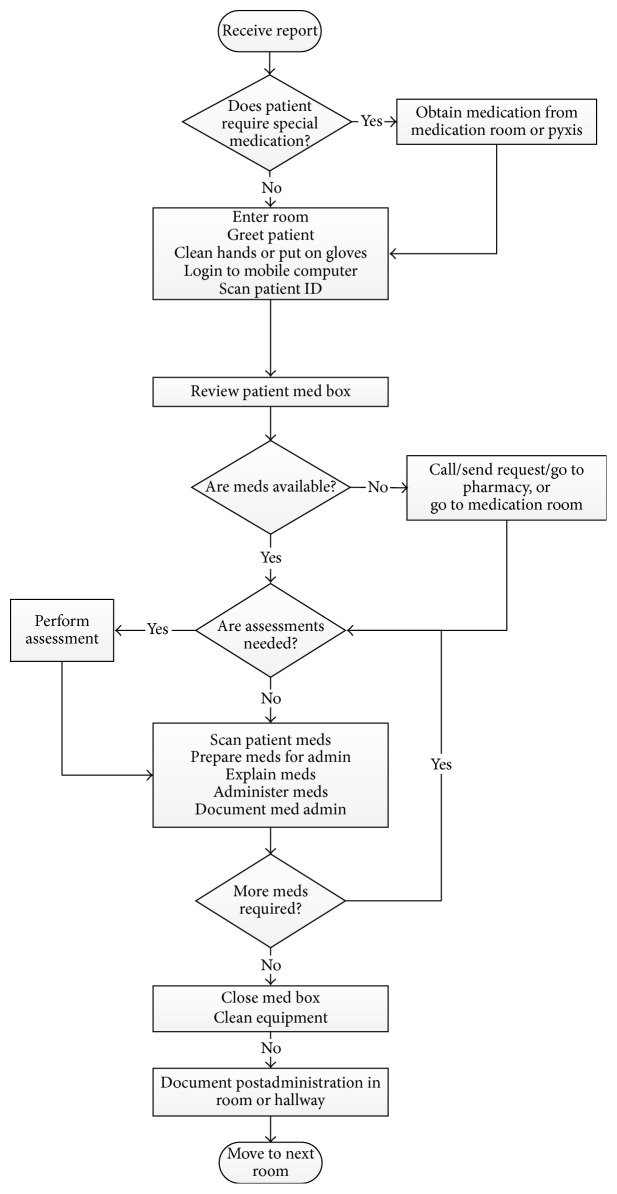
Unbundled MAP workflow.

**Figure 3:**
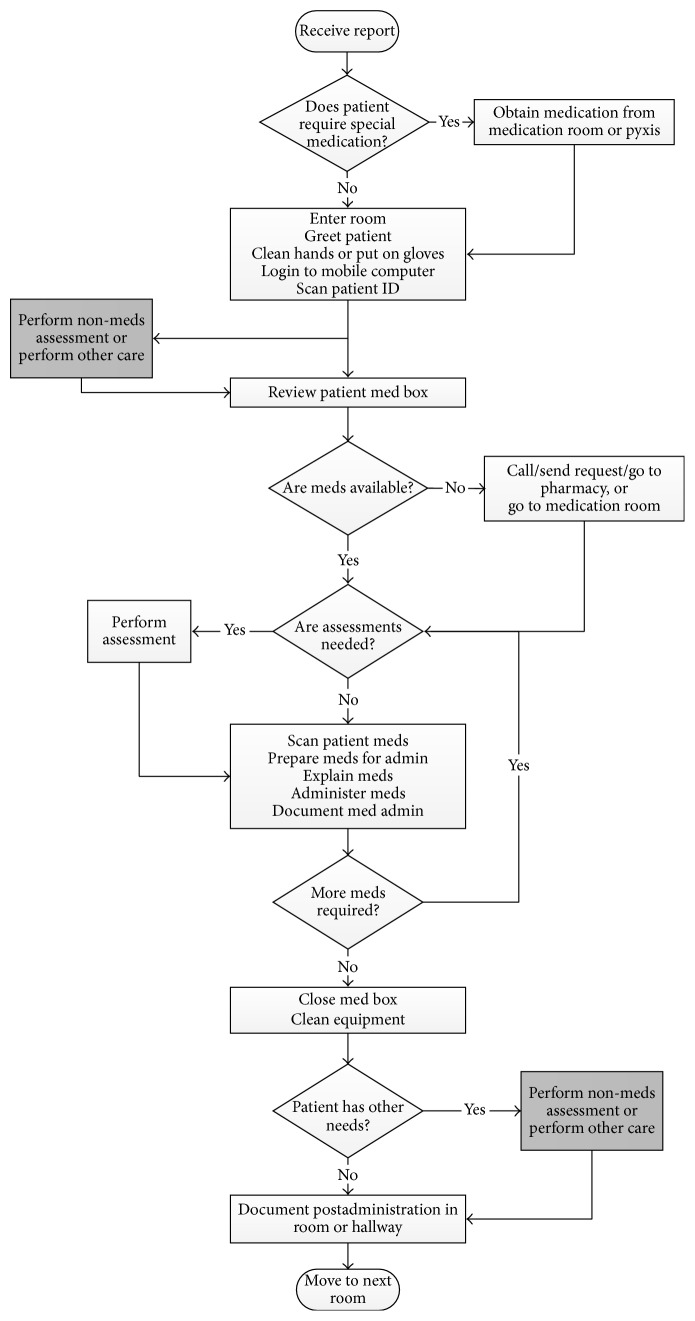
Bundled MAP workflow.

The first step of the data analysis was to develop process maps of the MAP. A process map is a pictorial representation of the sequence of actions that comprise a process. They were developed to provide baseline information on how the MAP was being performed and to understand its process characteristics. Analysis of the MAP data revealed that there were generally two distinct workflow processes: unbundled and bundled. In the unbundled workflow, an RN engaged in the MAP by performing only MAP tasks during a care episode (Figure 2). In the bundled workflow, an RN completed medication administration together with other patient care responsibilities during the care episode (Figure 3).

As shown in Figure 2, the unbundled MAP workflow typically started when the RN received and reviewed reports of patients she/he was assigned for that shift. After reviewing the reports, the RN then determined if any of her/his patients required special medications that were located in the medication room or Pyxis (a medication dispensing machine located inside the medication room). If so, she/he would retrieve them. The RN then visited each patient one at a time. The care episode or observation began when the RN entered the patient's room. This was often followed by a combination of tasks such as greet patient, clean hands or put on gloves, log in to mobile computer, and scan patient's ID. After reviewing the patient's medication list, the RN reviewed the medication box that was located in the patient's room to determine if all of the needed medications were available. If not, she/he needed to call the pharmacy, send a request to the pharmacy through the computer, or go to the pharmacy or medication room. These events were considered patient-driven interruptions; an interruption is an event that requires the RN to momentarily break away from his/her current task. If the medications were available, then the RN would check to see if any of the medications to be given required an assessment (i.e., a pulse for a patient receiving Digoxin). Once assessments were done, if applicable, the RN then proceeded with the medication administration tasks: scanning patient medications, preparing medications for administration, explaining medications to the patient, administering the medication, and documenting the medication administration. The medication administration tasks were repeated for however many medications the patient needed. Once the RN completed the medication administration tasks, she/he then closed the medication box and cleaned her/his equipment. This was followed by the postadministration medication documentation task. Some RNs performed this task while they were still inside the patient's room, while others preferred to do it in the hallway. The care episode or observation ended when the RN moved to the next patient's room.

As highlighted in Figure 3 for the bundled MAP workflow, the key difference between the unbundled and bundled workflow was the added “nonmedication assessment or other care” tasks. These included tasks such as changing a wound dressing, assessing the physical and mental capacity of the patient (Rule #9, Table 1), or providing assistance with dietary needs. It was observed that these nonmedication tasks could take place before, during, or after medication administration.

#### 2.4.2. Process Characteristics

Table 2 provides a summary of the differences between unbundled and bundled observations. Of the 305 observations, there were almost twice as many bundled observations as there were unbundled ones (203 versus 102). Bundled observations took nearly twice as long to complete (26 versus 14 minutes). The contrast in standard deviations indicated that the bundled observation was highly variable, with some observations taking nearly two hours to complete. Lastly, bundled observations were more likely to be associated with interruptions (patient-driven or time-driven) compared to unbundled observations (92% versus 82%). That is, the bundled MAP workflow had a 92% chance of having one or more interruptions. Patient-driven interruptions were defined as those that were triggered by patient-related care, whereas time-driven interruptions were triggered by other sources (considered as random events) (Table 3).

**Figure 4:**
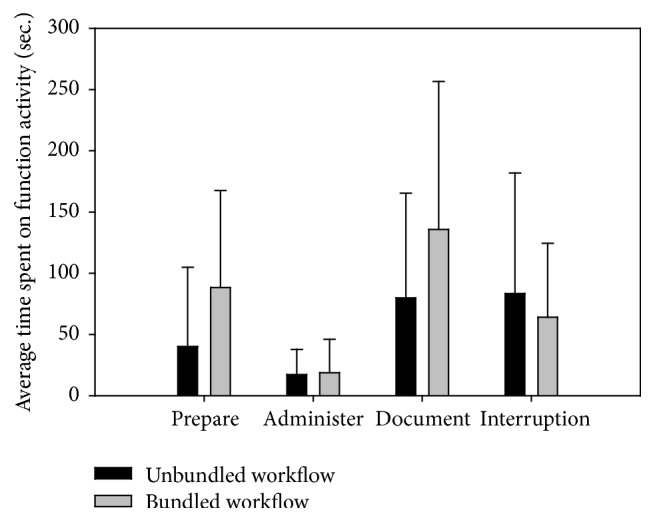
Average MAP task time (error bars indicate one standard deviation).

Figure 4 shows the difference in the average amount of time between unbundled and bundled MAP workflows for an RN performing specific MAP tasks, for example, prepare medication, administer medication, and document medication. Additionally, Figure 4 shows the difference in the time spent on interruptions for the unbundled and bundled MAP workflows. The error bars in the graph denote one standard deviation. Results indicated that RNs spent more time preparing and documenting medication for the bundled MAP workflow. The time spent administering medications was comparable between the two MAP workflows. While the unbundled MAP workflow had a lower chance of incurring interruptions, the duration of interruptions was slightly longer than those that occurred during the bundled MAP workflow. This may indicate that patient-driven interruptions are more time consuming.

**Figure 5:**
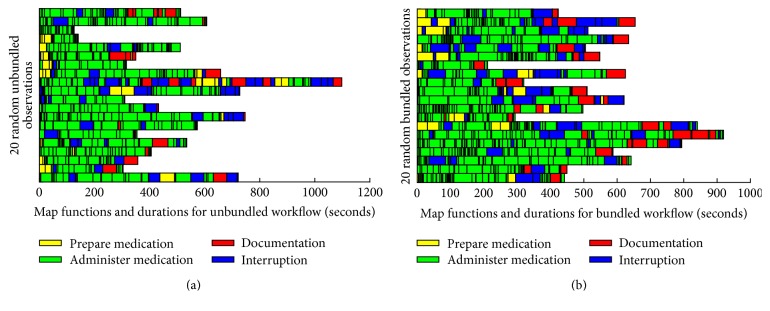
Timeline belt: (a) unbundled workflow; (b) bundled workflow.

To assess MAP workflow fragmentation, a timeline belt comparable to that proposed by Zheng et al. [23] was developed for both the unbundled (Figure 5(a)) and bundled (Figure 5(b)) MAP workflows. Twenty (20) observations were randomly selected for each workflow type. Each timeline belt or row in Figure 5 represents one MAP workflow observation. For each observation, MAP tasks and interruptions were mapped into one of four functions: prepare medication (yellow), administer medication (green), document medication (red), and interruption (blue). The length of a colored segment is proportional to the duration (in seconds) of the task or interruption. Thus, the longer an observation is the longer the timeline belt is, and the more fragmented an observation is, the more colored segments there are. As visually depicted in Figure 5, RNs engaged in a bundled MAP workflow switched between MAP functions and tasks more frequently (higher number of segments), experienced more interruptions (more blue segments), and took longer to care for patients (longer timeline belt).

## 3. Model Development and Validation

This section presents the methodology utilized in developing the MAP simulation model that explicitly models the observed bundled and unbundled workflow processes. This is accomplished via the incorporation of the statistical model discussed in Section 3.1. In Section 3.2, the design and functionality of the simulation model are explained in detail, followed by the discussion of the model input parameters and values. Lastly, the model validation procedure and results are discussed. The potential use of the validated simulation model is demonstrated in Section 4 where it is used to assess redesign interventions.

### 3.1. Statistical Model

Empirically, the bundled versus unbundled MAP workflow processes seemed to be related to the RN's level of experience; that is, the more experienced the RN, the more she/he bundled care tasks to enhance organization and efficiency. We, therefore, tested the hypothesis that nurses' work experience would have a significant impact on the number of patient-driven interruptions in unbundled and bundled cases. Since each RN was observed multiple times during the study, a mixed Poisson model was used to estimate the expected number of patient-driven interruptions during each observation. This model incorporated the random intercept into the model to accommodate the variations of measures within and between RNs. Using the mixed Poisson also accounted for overdispersion (conditional variance exceeds the conditional mean). Initially, we included all RN demographic variables in the model as covariates to assess the impact of moderator variables. A stepwise model selection procedure was used to remove the nonsignificant variables based on the *p* values. In addition, we also removed the variables which failed to meet the convergence criteria during the approximation procedure. In the bundled observations (Table 4), the significant variance of random intercept indicated that heterogeneity existed in terms of RN patient-driven interruptions. The significant negative effect of “years of work in hospital” implied that experienced RNs were able to reduce the number of patient-driven interruptions. In contrast, there was no significant variation among RNs or effect of “years of work in hospital” in the unbundled observations. The analysis was conducted using SAS/STAT 9.2 [24].

### 3.2. Computer Simulation Model

Many health care studies have utilized discrete-event simulation (DES) to model the operation of a system as a discrete sequence of events in time. The MAP workflows reflected a discrete set of events and were best modeled using DES. However, the RNs were not a homogenous set of agents with identical characteristics and this had an effect on their MAP workflows. In particular, the RNs' number of years worked in the hospital was correlated with the number of patient-driven interruptions for bundled workflows. For this reason, it was necessary to model RNs as individual agents with their own characteristics.  Pseudocode 1 provides a high-level algorithmic view of the simulation model, which was implemented using Netlogo [25]. We chose Netlogo because of our prior experience with it.

As illustrated in Pseudocode 1, the simulation model has three key procedures:* setup*,* simulate*, and* do-task*. The* setup* procedure performed all the preliminary steps before the workflow is simulated. In this procedure, the model initialized the variables, drew the layout of the units (to show location of RNs), generated a user-specified number of RNs working on a unit and patients to be assigned to each RN (the ratio of RNs to patients is based on collected data), and assigned specific attributes to each RN. The model simulated RNs' movements according to the actual study clinical unit floor layout and patient room numbers. This was done to track the total RN walking distance as a proxy for fatigue, which has long been considered a contributory factor in patient care errors [23]. The RN attributes that were explicitly modeled included the workflow type (i.e., bundled or unbundled) and the “years of work in hospital.” As discussed, the characteristic “years of work in hospital” was used to estimate the number of patient-driven interruptions each RN would encounter per observation. Time-driven interruptions were calculated per 10-minute intervals using the probability determined from data.

The* simulate* procedure was responsible for controlling the program flow. It maintained a queue of RNs to simulate and RNs were selected from this queue based on the timing of the events to be simulated. For each RN, it determined if the RN had completed all the tasks for an observation. If so, the RN was moved to the next room (i.e., observation/patient). If not, it assigned the next task to the RN and the time it took to complete that task. This information was then placed in the priority queue and subsequently drawn when it got to the front of the queue. The task processing times were determined using best-fit distributions and parameters (determined based on sample data). Thus, the simulation model is a stochastic model due to the randomness in task processing times and number of interruptions. The* simulate* procedure ended when all RNs had completed their assignments.

The* simulate* procedure relied on the* do-task* function to determine the next task within the unbundled or bundled workflow. The “conditional” term in the* do-task* function denoted a decision point in the workflow (represented by diamond shapes in Figures 2 and 3). When a conditional was encountered, the model determined the branch it followed based on the observed probabilities. Thus, while MAP activities were structured based on the workflows, there was significant workflow variability due to a number of conditionals (i.e., special medications, medication availability, medication-related assessments, and other care).

**Figure 6:**
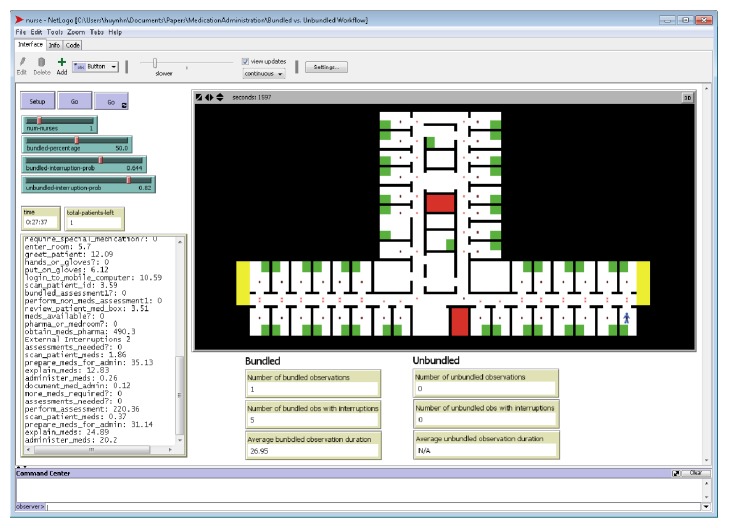
Graphical user interface of MAP simulation model.

Figure 6 shows a screenshot of the developed MAP simulation model. The graphical user interface includes the “setup” button that calls the* setup* procedure and the “go” button that calls the* simulate* procedure. Additionally, the GUI includes sliders that allow users to specify the number of RNs to simulate, the probability that an RN follows the bundled workflow, and the probabilities that they will encounter interruptions in the unbundled and bundled workflows. When the model is running, the GUI shows which rooms the RNs are in, their current activities, and relevant statistics.

### 3.3. Input Parameters

The simulation model uses a number of input parameters. The primary input is the list of tasks associated with unbundled and bundled workflows.  Algorithm 1 provides a portion of the codes we implemented to model the bundled workflow. Each row in Algorithm 1 is an “edge” in the workflow graph. The first item is the “from” task of the edge. If there is no second item in the row then the first item in the next row is the “to” task. If the task ends in a “?” then it is a conditional, to be executed at runtime. If the conditional statement evaluates to true (based on the observed probabilities) then we perform the second task in the row; otherwise, we perform the third task in the row. The task durations were drawn from the Gamma distribution with parameters *α* and *λ*, where *α* = mean *∗* mean/variance and *λ* = mean/variance. The durations of the commonly encountered bundled tasks are shown in Table 5; there are over 60 tasks in each of the two workflows; thus, not all tasks are shown.

Values related to the number of patients assigned to a nurse, the number of patient-driven and time-driven interruptions, and the number of medications needed by a patient were drawn from a discrete empirical distribution constructed from the observed data.  Table 6 shows the cumulative distribution function (CDF) values used by the simulation model to determine the values for these parameters. That is, a random number between 0 and 1 is generated and the CDF is used to determine the value for the corresponding parameter.

### 3.4. Model Validation

Model validation is the process of ensuring that the simulation model behaves in the way it was intended according to the modeling assumptions made. The MAP model validation was achieved by comparing the model's observation duration with the actual MAP observation duration. Netlogo's Behavior Space tool was used to generate 1,000 observations (i.e., replications) and each observation was run until the nurse completes MAP for all of her/his assigned patients. Note that the duration of each replication is dependent on a number of random factors, including how many patients are assigned to the nurse and how many medications are needed by the patients. The model's average observation durations were compared against the actual average observation durations. Since the observation durations are not normally distributed, the Wilcoxon Rank-Sum Test was used to test the null hypothesis that the distribution of observation durations generated by the model was the same as that of the actual observation durations. The Wilcoxon test, using the statistical software R [26], yielded a *p* value = 0.5341 for unbundled observations and a *p* value = 0.7629 for bundled observations, and the null hypothesis could not be rejected.

## 4. Assessment of MAP Redesign Interventions

The validated MAP simulation model was used to assess the impact of two hypothetical redesign interventions in a simulated setting: (1) intervention 1 increased RNs' use of an unbundled MAP workflow and (2) intervention 2 reduced MAP patient-driven interruptions. For intervention 2, the time-driven interruptions were not considered because they are random and RNs do not have control over them.

**Figure 7:**
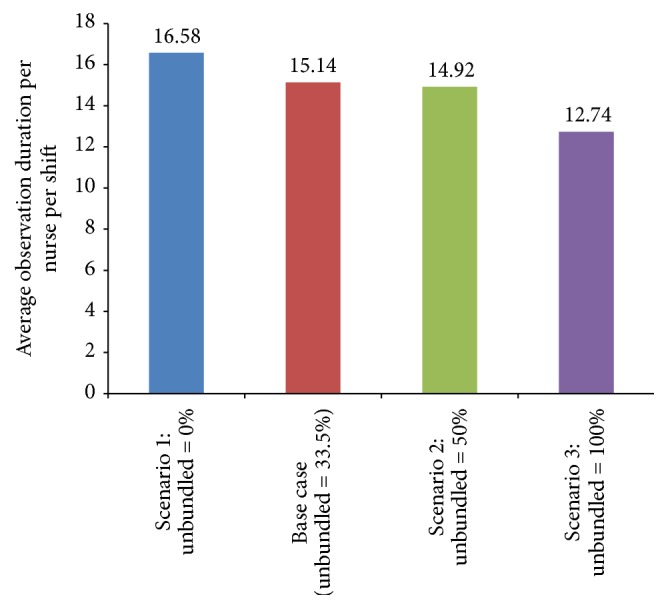
Impact of intervention 1 on average (mean) observation duration in minutes (note: base case and scenario 2 have a mixture of unbundled and bundled observations).

To assess the impact of intervention 1, the input to the MAP model was modified to have different percentages of RNs follow the unbundled workflow, that is, 0%, 33.5% (base case), 50%, and 100%. The experimentation was conducted using 1,000 replications and each replication was run until the nurse completes all of her/his assignments.  Table 7 provides a summary of the results for intervention 1, and Figure 7 illustrates the impact of these scenarios on the average observation duration per RN per shift. The results indicated that as the percentage of RNs following the unbundled workflow increased, the average number of interruptions and observation duration decreased.

To assess the impact of intervention 2, the input to the MAP model was modified to assume a reduction of 10%, 20%, and 30% in patient-driven interruptions for both unbundled and bundled workflows. Tables 8 and 9 provide a summary of the results for the unbundled and bundled workflow, respectively. As shown in Table 8, for unbundled workflow, the results indicated that as the probability of interruptions decreased, so did the interruption counts and observation duration. This result implied that if RNs could avoid situations that lead to interruptions, they would be more efficient. A similar result was observed for bundled workflow (Table 9), except that the average observation duration did not follow a decreasing trend. This was most likely due to other nonmedication tasks incorporated into the bundled workflow. Also, the patient-driven interruption time for bundled workflow tended to be lower than that of unbundled workflow. Thus, the fewer number of patient-driven interruptions had less of an impact on observation duration for bundled workflow than it did for unbundled workflow.

## 5. Discussion

Study findings indicated that there was a lack of consistent MAP workflow among RNs. Two predominant MAP workflow patterns, unbundled and bundled, emerged from the data. However, even within these two workflow patterns, additional variations were observed. For example, the number of patient-driven interruptions was associated with an RN's years of work in the hospital. Hence, it is possible that other RN characteristics might play a role in RN MAP workflow and additional research is needed in this area.

MAP timeline belts suggested that the bundled workflow is not desirable due to a higher incidence of MAP task switching (i.e., workflow fragmentation) and interruptions. Previous research findings have shown that frequent task switching often increases physical activities that can result in a higher likelihood of cognitive slips and mistakes that can lead to performance error [23]. Study qualitative findings from tape recorded comments pointed to a number of interruptions that were likely to occur from “other care” when RNs followed a bundled MAP workflow pattern. For example, RNs engaged in a bundled MAP workflow pattern often responded to patient requests for dietary assistance and other care requests such as toileting assistance (Table 10). While we classified these patient requests as interruptions, we recognize that they are necessary or essential to the patients' wellbeing. Moreover, we recognize that RNs cannot seek to be more efficient at the expense of the quality or completeness of care. Nevertheless, these “other care” interruptions raised a variety of nursing practice questions about the safety impact of the bundled MAP workflow pattern on medication administration error outcomes and other practice considerations such as use of aseptic technique in medication administration. Additional research is needed to address these and other emergent questions.

## 6. Conclusions

This study developed a computer simulation model to assess the performance of nursing unbundled versus bundled MAP workflows. It extended our previous work in several areas: (1) the iMedTracker was modified to work on iPad devices instead of iPod Touch to minimize scrolling and thereby facilitate the recording of live MAP observations, (2) data collection took place in an actual hospital setting instead of an academic simulation laboratory and thereby provided more realistic RN characteristics and MAP workflow observations, (3) the MAP agent-level DES simulation model was modified to incorporate RN and MAP characteristics to account for patient-driven and time-driven interruptions, and (4) statistical modeling techniques were used to compliment development of the MAP computer simulation model. While our methods and tools were validated in this study, key limitations should be considered when reviewing study results. These included (1) one study hospital, (2) only 2 medical units, (3) small number of RN participants (*n* = 17), (4) lack of patient contextual data, (5) small number of MAP observations (*n* = 305), and (6) data collected only during morning shifts of Mondays, Wednesdays, and Fridays. These limitations prohibited us from conducting an actual process redesign with the stakeholders. We plan to address these shortcomings in a larger proposed field study that is currently under review. Furthermore, in future work, we plan to develop an agent-based model of MAP that simulates the complex behavior of RNs through the use of agents. Specifically, we wish to model the autonomous behavior of the nurse agents and the interactions between the agents themselves and their environment. To accomplish this goal, we are currently expanding the capabilities of the iMedTracker to provide data for the agent-based model development and validation.

## Figures and Tables

**Pseudocode 1:**
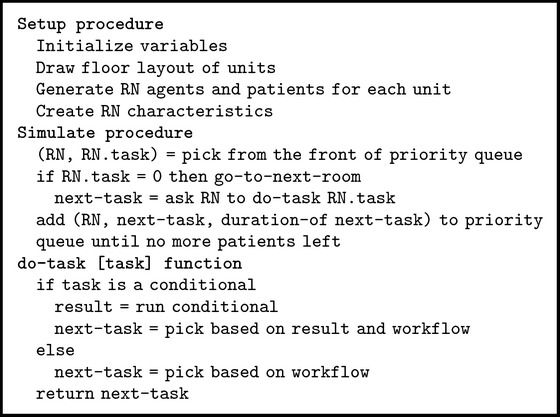
MAP computer simulation model high-level pseudocode.

**Algorithm 1:**
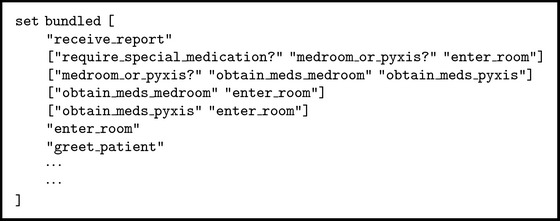
Bundled workflow data input to simulation model.

**Table 1:** MAP observation rules.

Rule #	Description
1	Each observation must begin with an “enter room” and end with a “move to next room.” The “enter room” should be entered only once when the RN first entered the room and should occur before “greet patient.” The “move to next room” should be recorded after the postdocumentation is done. Between the “move to next room” and “enter room” tasks, if the RN is performing activities related to the medication, then record all such activities.

2	The “other care” task is for direct/hands on patient care such as changing a wound dressing.

3	The “assess patient for other needs” task is for the final check of the patient's well-being; for example, is there anything else I can do for you?

4	For intravenous medication (IV) administration, the pre/post line flushes are part of administration.

5	For med administration, activities performed away from the bedside should be recorded as “prepare for medication admin” and at the bedside should be recorded as “administer medications.”

6	When the RN does not complete med administration—moves to the next patient—then returns to the original patient to complete med administration, this is considered a new observation and thus a new set of “enter room” and “move to next room.”

7	If the patient does not swallow oral medications, the end time of “administer medication” should not be recorded.

8	Products given to the patient during med administration that are not reviewed/documented on the patient's med record, for example, Orajel, are not considered medications.

9	The “perform assessment” task is for both mental and physical assessment.

10	Activities at the computer between scanning of meds are considered documentation.

11	Whenever the RN leaves the room during the med admin process, specify one of the “leave room” reasons, not “move to next room.”

12	The “review patient computer record” task is used when the RN is looking at the computer screen listing of prescribed medications. The “review patient medication box” task is used when the RN is looking at the medications. Be careful when recording these tasks as they are next to each other.

13	When an RN goes to the medication room, change location accordingly and record the task as “obtain medications: medication room.” If the RN accesses the pyxis, then record the task as “obtain medications: pyxis” while the other task is still active. Stop the “obtain medications: pyxis” task when the RN closes the pyxis, and stop the “obtain medications: medication room” task when the RN exits the medication room.

**Table 2:** Characteristics of unbundled versus bundled observations.

	Unbundled	Bundled
Number of observations	102	203
Number of observations with interruptions	84	187
Probability of interruptions	0.82	0.92
Average duration of observation (minutes)	14.11	26.06
Standard deviation of observation duration (minutes)	12.378	90.95

**Table 3:** MAP workflow interruptions.

Patient-driven	Time-driven
Leave room to obtain supplies	Leave room due to bed alarm
Leave room to locate medication	Staff inquiry
Leave room due to patient request	Personal time
Leave room to assist patient to bathroom	Answer phone call
Leave room due to family request	Make phone call
Patient inquiry	Computer battery issue
Family inquiry	Unable to scan med
Medication follow-up	Other interruptions
Obtain supplies from utility room	
Obtain supplies from medication room	
Obtain supplies from kitchen	
Obtain supplies from front RN station	
Obtain supplies from back RN station	

**Table 4:** Parameter estimates and standard errors of Poisson mixed model.

Parameter	Bundled model	Unbundled model
Random effect variance	0.143 (0.052)^*∗*^	0.170 (0.102)
Years of work in hospital	−0.048 (0.021)^*∗*^	0.004 (0.028)

^*∗*^ *p* value < 0.05. All models are adjusted for race, gender, years of licensure, and years of practice.

**Table 5:** Duration of bundled tasks.

Task name	Average duration (sec)	Std. dev. (sec)
Clean hands (upon entering room)	5.08	10.14
Put on gloves	13.82	6.38
Enter room	2.34	3.32
Greet patient	3.38	6.37
Login to mobile computer	7.51	4.65
Review patient computer record	17.65	25.99
Scan patient ID	6.52	4.90
Perform assessment	63.75	78.65
Review patient med box	17.04	16.42
Scan patient meds	4.24	6.16
Document med admin	6.43	7.64
Administer meds	68.94	88.72
Other care	61.65	99.21
Explain meds	13.63	14.35
Prepare meds for admin	34.47	37.19
Close medication box	4.67	6.66
Clean equipment	19.71	19.41
Clean hands (upon leaving room)	5.87	9.88
Document post administration (in hallway)	148.67	118.42
Document post administration (in patient's room)	76.06	106.43
Obtain meds from pyxis	42.92	27.92
Obtain meds from medication room	52.19	39.69
Obtain meds from pharmacy	489.00	162.53

**Table 6:** Simulation model parameters.

Model parameter	Discrete cumulative distribution function
Number of patients assigned to nurse (1,2,…, 11)	DISC (0,0.23171,0.14634,0.08537,0.14634,0.17073,0.08537,0.10976, 0.01219, 0,0, 0.01219)

Number of patient-driven interruptions (for unbundled workflow: 1,2,…, 11)	DISC (0,0.37333,0.22666,0.22666,0.10666,0.01333,0.01333, 0.02670,0, 0,0, 0.01333)

Number of patient-driven interruptions (for bundled workflow: 1,2,…, 11)	DISC (0,0.225,0.2375,0.21875,0.1375,0.09375,0.04375,0.00625,0.00625, 0.0125,0.0125,0.00625)

Number of time-driven interruptions (for unbundled workflow: 1,2,…, 6)	DISC (0,0.61165,0.26214,0.05340,0.04854,0.01456,0.00971)

Number of time-driven interruptions (for bundled workflow: 1,2,…, 8)	DISC (0,0.57271,0.27069,0.09620,0.04251,0.01119,0.00224, 0.00224, 0.00224)

Number of medications needed by a patient (1,2,…, 16)	DISC (0, 0.032787,0.2,0.118033,0.12131,0.10492,0.07213,0.09836,0.06885, 0.02951,0.05246,0.02951,0.01311,0.01311,0.01639,0.01311,0.01639)

**Table 7:** Results of intervention 1.

Unbundled percentage (%)	Average number of interruptions per RN per shift (95% CI)	Average observation duration per RN per shift, min (95% CI)
0	10.48 (10.24 to 10.71)	16.58 (16.34 to 16.82)
33.5 (base case)	9.15 (8.93 to 9.37)	15.14 (14.92 to 15.36)
50	9.02 (8.81 to 9.24)	14.92 (14.70 to 15.14)
100	7.17 (7.0 to 7.34)	12.74 (12.55 to 12.92)

^*∗*^Confidence intervals were calculated using Kelton et al.'s method [18].

**Table 8:** Results of intervention 2 for unbundled workflow.

	Probability of incurring interruption	Interruption counts per RN per shift (95% CI)	Average observation duration per RN per shift, min (95% CI)
Base case	0.82	7.46 (7.28 to 7.63)	13.66 (13.23 to 14.09)
10% reduction	0.738	6.75 (6.58 to 6.92)	13.38 (12.95 to 13.80)
20% reduction	0.656	5.86 (5.70 to 6.02)	13.30 (12.88 to 13.72)
30% reduction	0.574	5.19 (5.05 to 5.34)	13.23 (12.81 to 13.65)

^*∗*^Confidence intervals were calculated using Kelton et al.'s method [18].

**Table 9:** Results of intervention 2 for bundled workflow.

	Probability of incurring interruption	Interruption counts per RN per shift (95% CI)	Average observation duration per RN per shift, min (95% CI)
Base case	0.92	10.15 (9.93 to 10.37)	18.40 (17.82 to 18.98)
10% reduction	0.828	9.73 (9.50 to 9.96)	17.80 (17.23 to 18.36)
20% reduction	0.736	8.26 (8.06 to 8.46)	18.02 (17.45 to 18.59)
30% reduction	0.644	7.49 (7.29 to 7.68)	17.19 (16.64 to 17.73)

^*∗*^Confidence intervals were calculated using Kelton et al.'s method [18].

**Table 10:** Nonmedication related sources of interruptions.

Category	Interruption source
Patient dietary assistance	Give patient requested food
	Get ice for patient
	Leave room to find apple sauce
	Leave room to dispose of patient's breakfast

Patient care assistance	Assist another patient
	Assist patient to the bathroom
	Get translator for patient
	Get towels from linen room
